# An Efficient and Secure Energy Trading Approach with Machine Learning Technique and Consortium Blockchain

**DOI:** 10.3390/s22197263

**Published:** 2022-09-25

**Authors:** Tehreem Ashfaq, Muhammad Irfan Khalid, Gauhar Ali, Mohammad El Affendi, Jawaid Iqbal, Saddam Hussain, Syed Sajid Ullah, Adamu Sani Yahaya, Rabiya Khalid, Abdul Mateen

**Affiliations:** 1 Department of Computer Science, COMSATS University Islamabad, Islamabad 44000, Pakistan; 2 Department of Information and Electrical Engineering and Applied Mathematics, University of Salerno, 84084 Fisciano, SA, Italy; 3 EIAS Data Science and Blockchain Lab, College of Computer and Information Sciences, Prince Sultan University, Riyadh 11586, Saudi Arabia; 4 Department of Computer Science, Capital University of Science and Technology, Islamabad 44000, Pakistan; 5 School of Digital Science, Universiti Brunei Darussalam, Jalan Tungku Link, Gadong BE1410, Brunei; 6 Department of Electrical and Computer Engineering, Villanova University, Villanova, PA 19085, USA; 7 Department of Information and Communication Technology, University of Agder (UiA), N-4898 Grimstad, Norway; 8 Department of Computer Science, University of Sialkot, Sialkot 51040, Pakistan

**Keywords:** consortium blockchain, branching, charging station, demand response, double spending, electric vehicles, energy trading, KNN, machine learning, vehicular energy network

## Abstract

In this paper, a secure energy trading mechanism based on blockchain technology is proposed. The proposed model deals with energy trading problems such as insecure energy trading and inefficient charging mechanisms for electric vehicles (EVs) in a vehicular energy network (VEN). EVs face two major problems: finding an optimal charging station and calculating the exact amount of energy required to reach the selected charging station. Moreover, in traditional trading approaches, centralized parties are involved in energy trading, which leads to various issues such as increased computational cost, increased computational delay, data tempering and a single point of failure. Furthermore, EVs face various energy challenges, such as imbalanced load supply and fluctuations in voltage level. Therefore, a demand-response (DR) pricing strategy enables EV users to flatten load curves and efficiently adjust electricity usage. In this work, communication between EVs and aggregators is efficiently performed through blockchain. Moreover, a branching concept is involved in the proposed system, which divides EV data into two different branches: a Fraud Chain (F-chain) and an Integrity Chain (I-chain). The proposed branching mechanism helps solve the storage problem and reduces computational time. Moreover, an attacker model is designed to check the robustness of the proposed system against double-spending and replay attacks. Security analysis of the proposed smart contract is also given in this paper. Simulation results show that the proposed work efficiently reduces the charging cost and time in a VEN.

## 1. Introduction

In this modern era, many technological advancements are introduced in various life fields, such as energy management, smart cities, E-Health, E-Education systems, etc. The concept of a global village brings different nations close and introduces a new concept of smart cities, which leads to the upgrading of traditional systems [1]. Conventional homes are converted into smart homes by equipping smart devices. Smart cities consist of multiple smart entities such as smart vehicles, smart homes, smart educational systems and smart hospitals. Similarly, traditional vehicles are replaced with smart and electric vehicles. Smart and electric vehicles are our main focus in this work.

Over the last few years, traditional vehicles have been equipped with the latest devices and functionalities. Therefore, it is assumed that all traditional vehicles will be transformed into smart and electric vehicles within a decade or two. In the last decades, the population of urban areas has increased, leading to serious issues such as the depletion of fossil fuels, drastic climate change, etc. According to [2], the number of autonomous vehicles is increasing rapidly, and in the near future, the market value of EVs will grow from $54.23 to $556.67 billion. Smart vehicle usage has many advantages. However, the increase in the number of smart vehicles raises many issues, such as road accidents, range anxiety and road congestion. In traditional transport systems, a central intermediary party is involved in the efficient data flow between charging entities and electric vehicles (EVs) [3]. However, the involvement of a third and central party creates many issues, e.g., lack of privacy, lack of trust and a single point of failure. Therefore, a decentralized system is required to resolve all these problems.

Blockchain technology has been introduced and integrated with different life fields to resolve the problems of existing centralized systems. It is a decentralized system that ensures cost reduction, security, trust and privacy among EV users [4]. In 2008, Satoshi Nakamoto introduced blockchain with the advent of Bitcoin [5]. With the drastic increase in population, the demand for energy usage has also increased by several orders of magnitude, which creates an imbalance between energy supply and demand. This imbalance of load leads to issues such as energy scarcity, irregular load shedding, increased electricity costs, etc. To tackle such issues, smart energy management is required, which helps to optimize the usage of scarce energy resources [6]. Furthermore, creating green, sustainable, clean smart cities is also needed in the current era. The energy sector has witnessed considerable developments in exploring new renewable energy sources (RESs) that help tackle the issues caused by usage of fossil fuels. The most common RESs include solar, wind and hydropower. Undoubtedly, RESs have immensely contributed to increasing energy generation with the balance of demand and supply. However, RESs also cause some issues; the most prominent are its volatile nature, which causes uncertainty for energy generation [7].

Many smart vehicles operating on electricity, termed EVs, establish a network among themselves while moving on roads. This network is termed a vehicular network (VN). A VN consists of multiple entities: charging stations, roadside units (RSU), EVs, etc. All these entities are linked together and perform various functions such as sharing road and weather information, charging of EVs via charging stations and saving important information in RSUs [8]. Various issues exist in VNs, such as lack of charging stations, trust issues among EVs, limited battery capacities of EVs, etc. Moreover, EV users are not comfortable charging their vehicles at a high cost/price and may travel long distances EV charging.

Optimal energy usage by EVs and establishing an equilibrium between supply and demand also need to be addressed. For this purpose, demand-response (DR) strategies are introduced, which help EV users adjust their energy demands according to charging time and price. These strategies also enable users to shift their energy demand from peak to off-peak hours, reducing the burden on energy grids and obtaining energy at low prices. Furthermore, DR aids in flattening load curves without deploying additional energy generators [9]. Moreover, great work has been done in integrating blockchain technology in the transportation sector. Similarly, this sector has also deployed DR strategies to help both EVs and energy grids. However, there still exist other issues such as privacy leakage, optimal pricing schemes and lack of user comfort [10].

This work aims to ensure the security of EV users and data immutability of transactions being generated and shared between EVs, RSUs and charging stations. Data storage issues are also solved in the proposed work. Moreover, EVs are charged at a low monetary cost by minimizing the burden of the charging entities. The nearest charging station to the EV is calculated using the K-nearest neighbour (KNN). Moreover, EV charging is done securely using blockchain technology and machine learning. Furthermore, EVs are charged according to their present state-of-charge (SoC). The time required to charge an EV is also calculated in the proposed work, and the data redundancy of transactions is also resolved. In a nutshell, the proposed work paves the way for efficient EV charging at a lower cost.

This paper is the extension of our conference paper [11]. The major contributions of this paper are given below.

### Contributions

- A blockchain-based secure energy trading system is proposed with minimum cost. The proposed system also achieves security and privacy by using blockchain.
- Transaction redundancy is tackled through a hashing algorithm. A hashing algorithm (SHA-256) is used as a tracer to remove redundancy.
- A machine learning algorithm, KNN, is used to calculate the shortest distance between a charging station and an EV.
- A reputation mechanism is proposed for the selection of EVs. This reputation mechanism also helps to avoid Sybil attacks.
- In the proposed system, energy aggregators are introduced as energy brokers that provide a significant way to find optimal charging stations for EVs with less energy consumption, calculate the time of charging and define the present state-of-charge.
- The proposed mechanism also calculates the energy required by an EV and presents the amount of energy available at the charging station. Moreover, DR is integrated with blockchain to manage EV demand and supply securely.

The organization of the paper is given below.

The related work and problem statement are mainly discussed in Section 2. The proposed system model is presented in Section 3. Simulation results are described in Section 4. Moreover, Section 5 presents a security analysis of the proposed system model and blockchain based attacks are discussed in Section 6. The conclusion of this work is presented in Section 7. Section 8 describes the future directions of proposed work.

## 2. Related Work

Nowadays, blockchain has gained tremendous attraction from industry and researchers and has become an emerging technology. However, some researchers have discussed the security risks and issues related to blockchain technology. The literature summary is given in Table 1.

In [12], the authors addressed issues related to power supply between the regions of a smart city. The authors proposed a dynamic complex network of grid-to-vehicle (G2V) energy networks. EVs can travel to multiple cities in this network. Thus, EVs act as energy transporters between multiple regions. However, the authors did not consider centralized energy storage points.

The authors of [13] identify the problem of inefficient charging and discharging and mention insecure energy trading between EVs. Therefore, they proposed a secure energy trading approach based on blockchain. Moreover, the authors proposed a different energy trading scheme in a blockchain-based system.

In [14], a charging guide strategy using a consortium blockchain system is proposed. The proposed work is aimed at dealing with the charging requirements of taxis. They use a practical Byzantine fault tolerance (PBFT) mechanism to achieve consensus in the proposed system. PBFT is also used to tackle the trust issue between multiple charging station operators. The charging guide model for the taxis in the proposed work is established using multi-objective optimization. The simulation results of the proposed work show that the passenger satisfaction is increased. In [15], the authors discussed different charging infrastructures and strategies in smart cities. In [16], the authors propose an energy trading model based on smart contracts and blockchain. They used a dynamic pricing strategy and a reverse-auction mechanism during trading. The proposed work not only benefits less-competitive power sellers but also reduces the electricity price. To tackle the trust issues among EVs, a decentralized trust management system based on blockchain is proposed. Another paper that addressed trust issues is [17]. In this system, received messages are verified by EVs using a Bayesian inference model. Upon receiving the message, a corresponding rating is generated for it. Using these trust values, RSU calculates trust value offsets for the EVs. However, the proposed work lacked discussion of collective trust management and privacy preservation.

In [37], the authors proposed an incentive scheme based on blockchain for energy trading. It provides efficient and secure energy trading between EVs and energy grids. Moreover, to enhance security, they proposed a reputation model and a secure distributed energy trading scheme for efficient energy trading. However, malicious entities are not considered in the proposed system. Moreover, in [18], the authors also proposed a contract-based energy trading scheme. In [19], the authors worked on secure and efficient data trading using consortium blockchain. The consensus mechanism in the proposed system is based on pre-selected nodes. A double-auction method is used; however, it consumes more energy because a large number of iterations are involved during the process. In [20], the authors propose an energy trading mechanism for plug-in hybrid electric vehicles (PHEVs). According to the proposed scheme, PHEV perform efficient charging with less energy cost. The authors also discuss the efficient communication of vehicles. However, the proposed scenario is expensive to implement in real life, and energy balancing is not considered. In [38], the authors introduced a hybrid peer-to-peer (P2P) energy trading system for energy markets.

In energy markets, a new concept related to EVs, grid-to-vehicle (G2V) and V2G, is introduced in [21]. This new concept proves that an energy grid is an advantageous entity. The EVs implement a bidirectional flow of communication and energy. Exponential growth in the number of EVs has occurred over the past years. This has led to environmental pollution. In [22], the security analysis is performed on the Brooklyn microgrid network, including the implementation of blockchain in the energy sector. The authors also proposed an energy trading model based on blockchain. An encryption scheme is used for the security of transactions. However, malicious operators and selfish mining are not considered. In [23], the authors addressed the issue of secure energy trading transactions between EVs.

In [24], the authors addressed a VN’s insecure energy management problem. The problem of centralized charging systems in VNs is also identified. A decentralized security model is used to resolve these problems. The proposed model is based on smart contracts and a lightning network. It resolves the issues of registration, authentication, scheduling and charging. In [25], the authors worked on the registration framework using blockchain. In [39], the authors used a deep convolution neural network (CNN) with blockchain for energy management.

The proposed model managed energy demand, storage systems, renewable energy and real-time electricity prices. In [26], the authors identify the issue of long delays in service response, low data-storage capacity, lack of trust between entities and high latency. Therefore, they proposed a blockchain-based intelligent, secure autonomous transportation system. There are two types of services used in this model: smart pay and smart share. However, the authors did not consider storage.

In [27], the authors address the issue of inefficient energy balancing and restricted battery capacities in an underwater sensor network (UWSN). Therefore, they proposed an improved metaheuristics-based clustering with the multi-hop routing protocol (IMCMR). A novel hybrid architecture network comprising blockchain and a software defined network (SDN) is presented in [28]. The proposed architecture has two parts: an edge network and a core network. Furthermore, the security issue is solved through the POW mechanism. However, the authors did not consider the efficient deployment of edge nodes. In [29], the authors address the controller selection problem. Therefore, they proposed an analytical network decision-making process (ANDP) that finds optimal controllers in the network. However, they did not consider scalability issues. In [40], an intelligent VN based on blockchain is proposed to deal with security threats and resolve trust issues. The proposed model communicates with vehicles and IoT devices without any security threat. However, the comfort of vehicle operators in a hassle-free network is not considered.

In [30], a blockchain-based, decentralized, distributed and secure storage management scheme is proposed in a VN. The proposed scheme increased the efficiency and performance of the network. However, during vehicle communication, channels are not reliable. Moreover, in [31], the authors also worked on a blockchain-based IoT traffic system. In [8], a decentralized trust management system based on blockchain is proposed. The proposed system uses a Bayesian interface model to validate the received messages. However, the large size of the data packets increases the message validation delay. This delay affects the efficiency of the network. In [41], the authors discuss P2P and V2G transactions of EVs in a VN. They proposed a blockchain- and smart contract-based EV trading model. The proposed decentralized power trading model considers the uncertainty and randomness of the charging and discharging of EVs. Furthermore, a reverse-auction mechanism is used to reduce the monetary cost of electricity. Further, the transportation sector has also stretched the number of vehicles running on roads. The ongoing scarcity of energy-generating sources has led the masses to face an imbalance between energy demand and supply [32], which has paved the way for other issues such as increases in energy prices, the absence of demand-response programs, etc. The increasing number of automobiles also adds to energy demand to a great extent.

In [42], the authors address the security and privacy problems in IIoT-based P2P energy trading networks. Therefore, they proposed a consortium blockchain-based secure energy trading system named ’energy blockchain’. However, it requires a high cost to maintain the energy blockchain with IIoT nodes. Further, the computational delay also increases. In [43], the authors proposed an incentive mechanism based on blockchain. In [33], the authors discussed storage problems. In [44], therefore, they proposed a blockchain-based trust management system. The proposed system resolves the issue of credibility of received messages.

In [45], blockchain technology is integrated with edge computing in a VN. These technologies are used for efficient V2G trading in the VN. To perform energy trading, a consortium blockchain is used. A contract-theory-based incentive mechanism is used that increases the involvement of users in the network. Resource allocation is resolved using a Stackelberg game and backward induction. The proposed work enables efficient V2G trading. However, the trading approach needs to be further discussed. In [34], the authors also proposed an incentive system for real-time renewable energy resources for vehicles.

For the efficient placement of charging stations, the authors of [35,46] considered different aspects such as driving range, voltage regulation, cost, etc. Each feature important for charging station placement is studied. The results show that the proposed work is globally acceptable and exhibits a small approximation error. It is a highly technical study and involves prioritization as well. However, this work lacks in providing a charging strategy for EVs, which is also necessary. In [36], the authors proposed an optimal pricing scheme for charging EVs with less cost. EVs also coordinate with each other under the proposed scheme. The proposed work lacks in dealing with the profits made by the charging stations. In [47], the authors addressed problems related to EV charging and discharging.

### Research Gaps

In recent years, many conventional vehicles have been transformed into smart and electric vehicles. These smart and electric vehicles come together and form a VEN. The communication of vehicles in these networks is beneficial. However, some major issues exist in VENs, such as scarcity of charging stations, inefficient energy management, load fluctuations on charging stations, etc. [12].

The authors of [48] put forward a method for finding the nearest charging stations. However, they encounter a major issue dealing with the geographical disparities related to the edge nodes. To ensure efficient energy trading, the authors of [49] develop the idea of sharing energy between users in a decentralized manner. However, issues of security, privacy, and trust are witnessed. Hence, user security and privacy preservation need realization in modern times. In VENs, energy aggregators also work as energy brokers in energy markets. Furthermore, centralized grids are used to charge EVs in traditional systems. This centralization leads to many issues, such as lack of trust, a single point of failure, and security and privacy concerns [13].

With the drastic increase in the number of EVs, the energy sector faces new challenges such as imbalanced load supply, voltage fluctuation, load-shedding, etc. Therefore, the integration of DR in VN becomes necessary because it efficiently manages the load supply and reduces the peak load. However, traditional DR systems involve third parties and lead to security issues, privacy leakage, increased cost, etc. [14]. Energy trading between EVs and charging stations also faces many problems, such as lack of location privacy and trust and imbalances between load and demand. Moreover, in [17], the authors worked on a blockchain-based decentralized energy trading approach in energy markets in which electricity is purchased at specified prices defined by utilities in the context of a one-sided market. Therefore, a DR approach is needed to help consumers establish a double-sided market in which utilities and consumers benefit equally.

## 3. System Model

**Figure 1:**
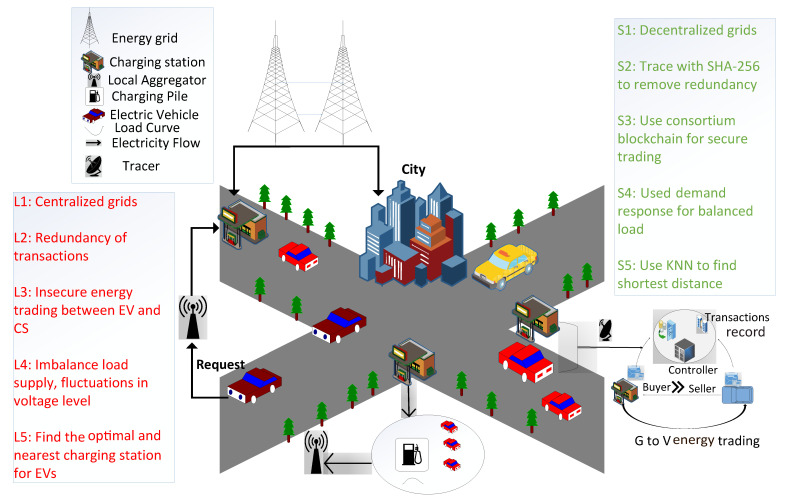
Proposed EV charging scenario.

Nowadays, energy trading activities ubiquitously take place in smart cities. However, in energy markets, secure energy trading is an important concern. Therefore, a secure energy trading mechanism based on blockchain is proposed. The proposed mechanism provides secure and efficient energy trading between charging entities, i.e., charging stations and EVs. The proposed model comprises EVs, charging stations, aggregators, energy grids, consortium blockchain, a tracer, and a DR mechanism, as shown in Figure 1.

The proposed system finds the nearest charging station for an EV. A machine learning algorithm, KNN, is implemented to find the nearest optimal charging station. When an EV needs to be charged, it sends the request, along with its current location, charging price and energy requirement, to the aggregator. According to the given location, the aggregator finds the nearest charging station for the EV and compares the amount of energy at the charging station to the EV’s requirement. If sufficient energy is not available at the charging station, the aggregator finds another charging station. In the proposed mechanism, the aggregator provides a list of the nearest available charging stations according to the location of the EV. The aggregator finds the required amount of energy that the EV needs to reach the charging station and also estimates the time required for the EV’s charging. The communication between EVs and charging stations is secure in the proposed mechanism because of the authentication process. A branching mechanism that deals with the problem of intensive data and reduces computational delay is also proposed in this system.

The proposed system model consists of the following components.

### 3.1. Electric Vehicle

EVs play a unique role in VENs because of their bidirectional energy trading capabilities. In the proposed system, EVs act as energy consumers that receive energy from different charging stations through aggregators. In the proposed scenario, the EV gets a list of charging stations from the aggregator and selects the nearest available charging station. The given list consists of available charging stations with their locations and the amount of energy at the selected charging station.

### 3.2. Charging Station

The charging stations obtain energy from the main power grids. These charging stations are also connected to aggregators placed in the respective areas. If these charging stations run out of electricity, they ask the energy grid to provide them with the required energy. All charging stations forward their updated energy information to the aggregators.

### 3.3. Consortium Blockchain

Consortium blockchain is a type of blockchain that can be used in a security system. In this blockchain, only specific, selected entities can maintain the access control of a system and perform only certain functions. Therefore, it is different from private and public blockchains. A consortium blockchain is used in the proposed scenario to resolve security issues.

### 3.4. Vehicle-to-Grid Energy Network

In traditional systems, centralized grids are used to provide energy to EVs and the city simultaneously. However, centralized grids have many challenges, such as a single point of failure, load imbalance, and security and privacy issues. Therefore, decentralized grids are proposed in the given system to overcome the above-mentioned problems. Decentralized grids help manage the load supply and demand in vehicular and residential areas.

### 3.5. Role of Aggregators

Figure 1 shows the communication of EVs and the aggregator. Aggregators act as energy brokers. When energy is required by an EV, it communicates with a nearby aggregator and sends an energy request to a nearby charging station. The aggregator finds a list of the nearest charging stations according to the requirements of the EV. It also confirms the EV’s energy price and charging requirement by using Algorithm 1. The proposed algorithm calculates the required energy of an EV and the energy consumed to reach the selected charging station. Every charging station has the ability for bidirectional communication. Therefore, it tackles energy flow according to market demand. Aggregators are the selected entities that manage access control during energy trading activities.

### 3.6. Role of Tracer

During energy trading, transactions are performed between EVs and charging stations. In this process, several transactions occur that create redundancy. The proposed model uses a tracer based on SHA-256 hashing to resolve this issue.

### 3.7. Energy Transportation by Electric Vehicles

V2G technology enables bidirectional energy flow in EVs. EVs have their own demand-response management (DRM) dynamics connected by the EV fleets. The dynamics of V2G energy networks are analyzed in this system. A dataset is used to describe the DRM dynamics in a specific region. There are three state variables for the description of DRM dynamics: (i) demand level of electricity, (ii) batter pool of SoC (iii) and the price of electricity.
**Algorithm 1:** Energy Trading Request
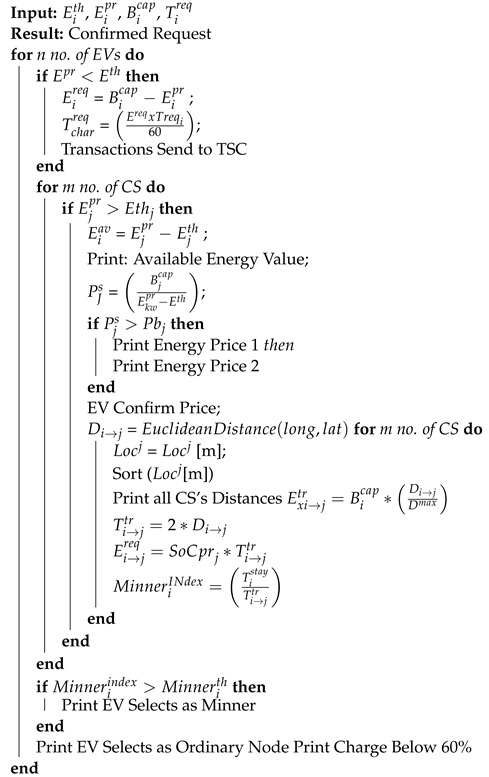


### 3.8. Demand Response

A typical DR scenario is used in this system. The proposed system includes major entities such as energy grids, charging stations, charging piles, EVs, etc. In the proposed work, the DR pricing strategy is used, which enables EV users to flatten load curves and efficiently adjust electricity usage. In DR, bidirectional communication takes place between entities, which enables the efficient flow of electricity and data between different entities. The DR mechanism shifts the energy demand of EVs from on-peak hours to off-peak hours. The given scenario efficiently manages the load curves. The demand-response scenario is discussed in Algorithm 2.
**Algorithm 2:** Demand Response
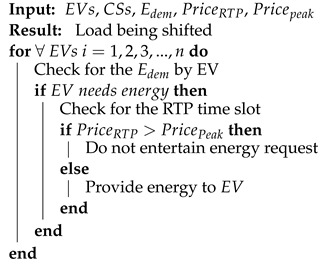


### 3.9. Finding Minimum Distance

The efficient charging of an EV depends on two major factors: distance from the charging station and the time taken to travel this distance. In the proposed work, we deal with the former factor, i.e., the distance to the nearest charging station. This distance is calculated using a KNN technique. When an EV requires energy to charge itself, it sends an energy request to the aggregator. The aggregator finds the nearest available charging station for the EV. This scenario efficiently reduces the expenses and traveling time. Algorithm 3 is used to select the charging station based on the shortest distance. The proposed algorithm also calculates the amount of energy and time that an EV needs to reach the selected charging station.
**Algorithm 3:** Selecting the Nearest Charging Station
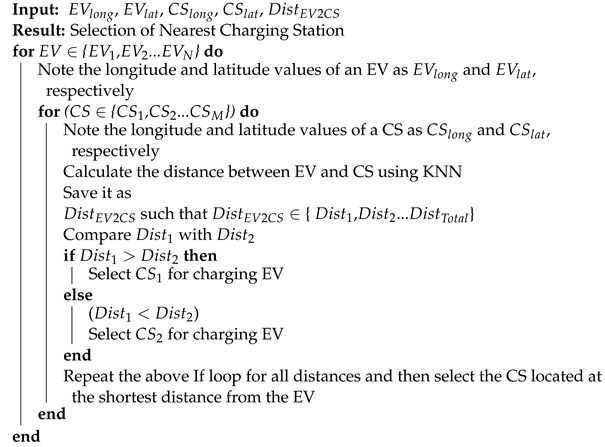


### 3.10. Selection of Charging Station Using KNN

KNN is a supervised learning algorithm in which learning is done from a labeled training dataset. According to the data, it finds the minimum distance between a specific point and all other points. It makes iterative predictions on the training data and the learning from the dataset.

This algorithm finds the shortest distance by using Euclidean distance, which is calculated using Equation (1), where $x_{1}$ and $x_{2}$ are longitude coordinates and $y_{1}$ and $y_{2}$ are latitude coordinates of two points.

(1) $$Dist_{Euclidean}=\sqrt{{\left(x_{2}-x_{1}\right)}^{2}+{\left(y_{2}-y_{1}\right)}^{2}}$$

Charging station selection depends on the shortest distance from the EV. The shortest distance is calculated using KNN. In the proposed model, the locations of charging stations are taken from the city of Oslo. This city has a large number of EV charging stations. The proposed algorithm lists the nearest charging stations according to the position of the EV.

The EV first selects the nearest charging station. If the selected charging station is busy, then the EV moves to the second-nearest charging station.

### 3.11. Trust-Factor-Based Reputation

In the proposed model, when two EVs send requests to aggregators simultaneously, the aggregator gives preference to the request of the EV with the higher reputation value. These reputations are based on trust factors. The Eigen trust reputation algorithm calculates the reputation values for the EVs. Basically, reputation is anticipation of the behavior of an EV based on its previous behavior and observations. An Eigen trust reputation algorithm provides a unique trust value to each EV based on the history of transactions made by each EV. It collects information from all peer EVs and calculates the trust value based on feedback. This algorithm provides unique trust values to each EV. This trust value depends on the transactional history of every EV.

The trust values either increase or decrease based on message credibility. When an EV sends correct data, the trust value increases, and vice versa. EV stakes are purely based on respective trust values.

This reputation mechanism is also used to avoid Sybil attacks. In a Sybil attack, the attacker generates multiple fake IDs to gain incentives. In the proposed system, every entity is registered and has its own reputation value; however, fake entities do not have any reputation value. Therefore, fake entities can be easily identified when a reputation system is implemented.

### 3.12. Registration and Authentication through Certificate Authority

**Figure 2:**
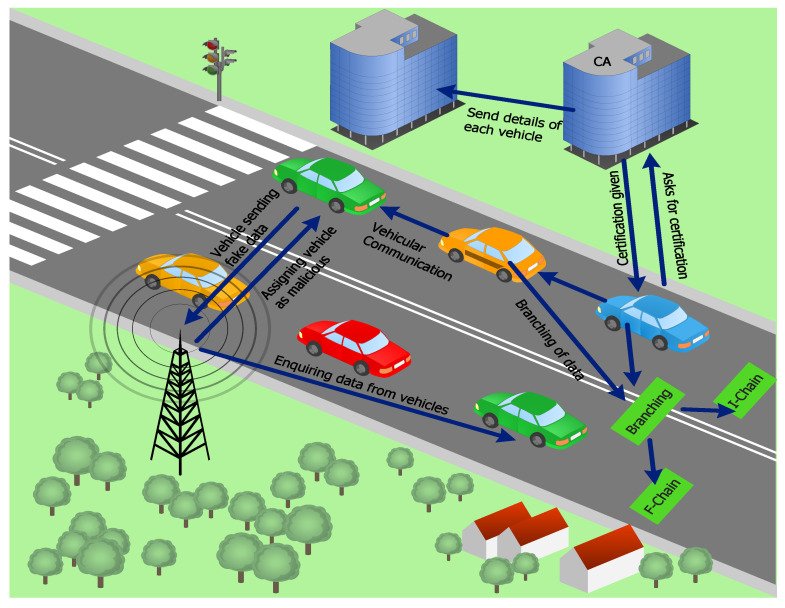
A scenario to detect malicious EVs.

Only registered EVs can communicate with other EVs, aggregators and the Certificate Authority (CA) in the network. In the proposed model, newly incoming EVs are first directed toward the CA. EVs make a request to the CA for entrance into the network. The CA provides a certificate to the EV that contains the EV’s real identification (id) and pseudonym id. These EVs collect data (e.g., road conditions, weather conditions, etc.) from their surroundings and pass it to the nearest RSU. The CA also grants authentication to EVs. Authentication resolves the issue of malicious EVs in the network. Figure 2, shows the scenario to detect the malicious EVs in the network. When EVs are authenticated through the CA, then they are free to participate in the network.

### 3.13. Branching of Data

In the proposed system model, a branching concept is used, in which the blockchain network comprises two further chains: I-chain and F-chain. Transactional data are divided into two parts because of the branching mechanism. Initially, all information related to EVs is added to the I-chain. However, based on malicious activities, EVs are added to the F-chain if they continuously send fake data to the RSU. The RSU node verifies the data of nearby EVs. If the data are fake, the RSU takes the real id of the EV from the cloud, revokes it from the network, and adds it to F-chain. Finally, the validated transactions and EVs are stored in the I-chain, and all malicious EVs are stored in the F-chain.

Therefore, the network becomes robust against the EVs’ intensive data, and computational delay is also decreased. When the delay is decreased, the performance of the network becomes efficient.

### 3.14. Payment to Charging Stations

Once an EV gets energy from the selected charging station, then the EV pays the charging station for the energy supply.

**Figure 3:**
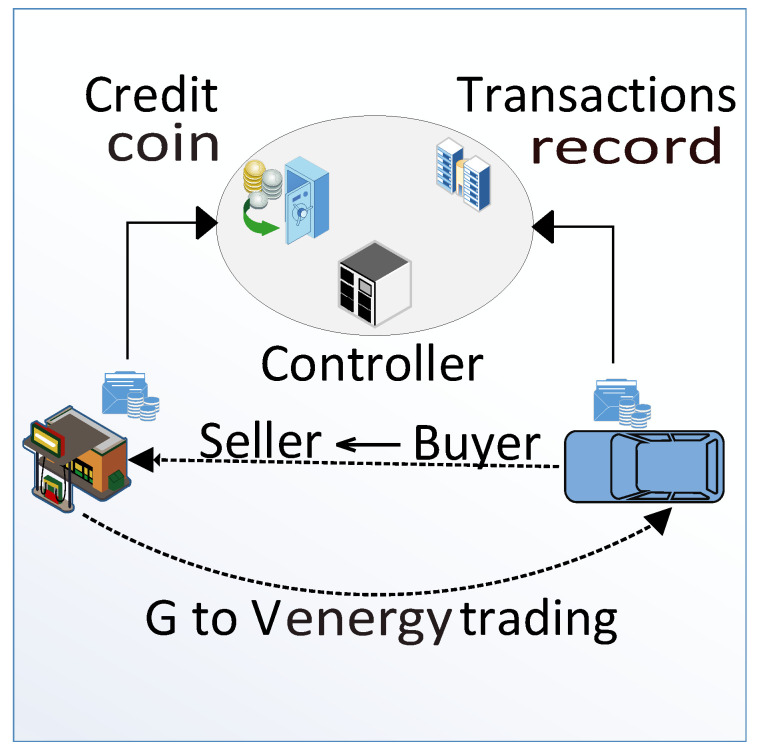
Charging payment.

This payment can be in the form of cryptocurrency or in the form of fiat money, i.e., USD, PKR, etc. Figure 3 shows the trading scenario between buyer and seller. A smart contract is established between both entities that verifies the amount of energy and energy price. All trading information is stored as transactions in the blockchain after it has been validated by the miners.

In Table 2, all limitations are mapped with proposed solutions.

## 4. Simulation Results

Simulation results are described in this section. This paper proposes an algorithm to find the optimal charging station for an EV. Moreover, a smart contract is implemented for secure energy trading. This smart contract is written in solidity. The simulation of a given smart contract is performed on RemixIDE.

Execution and transaction costs of the smart contracts are considered for evaluation. The conversion of ether into gas is taken from [50]. Moreover, for the testing and validation of smart contracts, MetaMask is used [51].

1 ether=207.6946 Gwei gas=378.839639 USD

The transaction costs and execution costs are directly proportional. They are calculated by Equation (2):

(2) Transaction Cost=Gas Used×Gas Price

**Figure 4:**
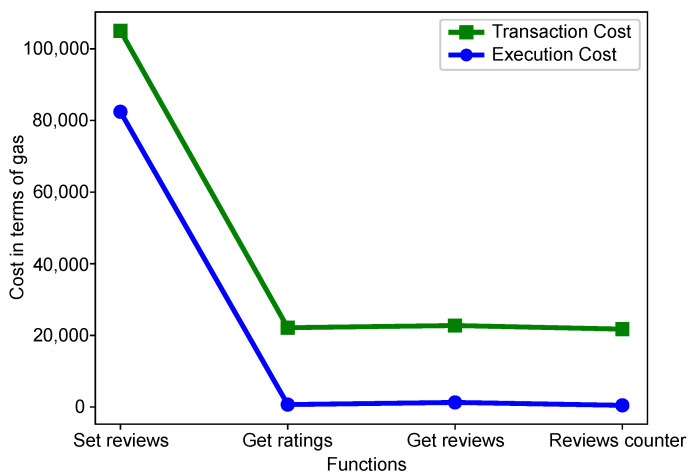
Gas consumption of smart contract.

Figure 4 shows the transaction and execution cost of smart contract functions. It can be seen that the transaction costs are greater than the execution costs. The transaction cost is the combination of deployment cost and function cost. Moreover, execution cost is only based on execution functions. This smart contract is used to assign the reputation values. This smart contract comprises the following functions: *‘Set reviews’, ‘Reviews counter’, ‘Get ratings’* and *‘Get reviews’*. The execution and transaction costs are highest for the *‘Set reviews’* function because at the occurrence of the first function all resources are initialized, which consumes more gas. This figure is the validation of L3 as shown in Table 2.

**Figure 5:**
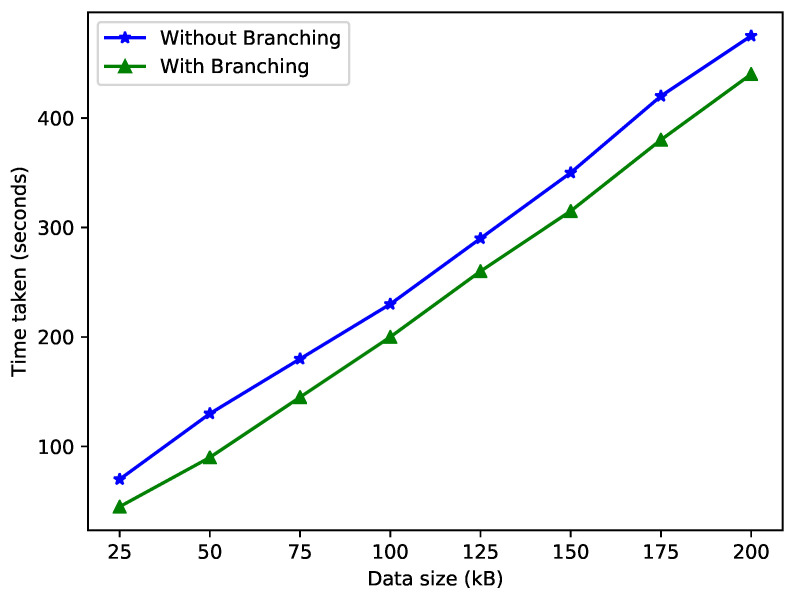
Time taken for data storage.

The concept of branching is used in this work. This phenomenon helps reduce the time taken for data storage and also decreases the computational delay. The data coming from malicious EVs are added to the F-chain, and only authenticated data are stored in the I-chain. Figure 5 shows the relation between data size and time with or without branching. When we store data on the same branch, it takes more time and space.

When data are stored in the blockchain through branching, it consumes less time because the data are divided into two types: fraud data and authenticated data. However, storing the data without branching consumes more time because the entirety of the data are stored in a single blockchain.

**Figure 6:**
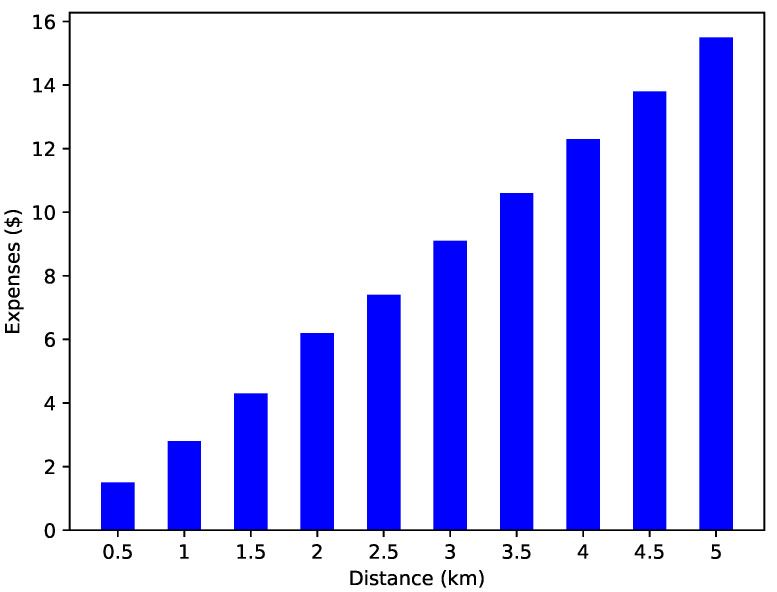
Expenses incurred while traveling.

Many factors play an important role in EV charging, with distance from the charging entity being one of them. EVs tend to select the charging entity that is near to them. Figure 6 shows the time required to reach the selected charging station. There is a proportional relation between time and distance. The time increased with respect to the distance. An increase in the distance also leads to increased expenses. Therefore, EVs tend to select the nearest charging entity to reduce expenses.

**Figure 7:**
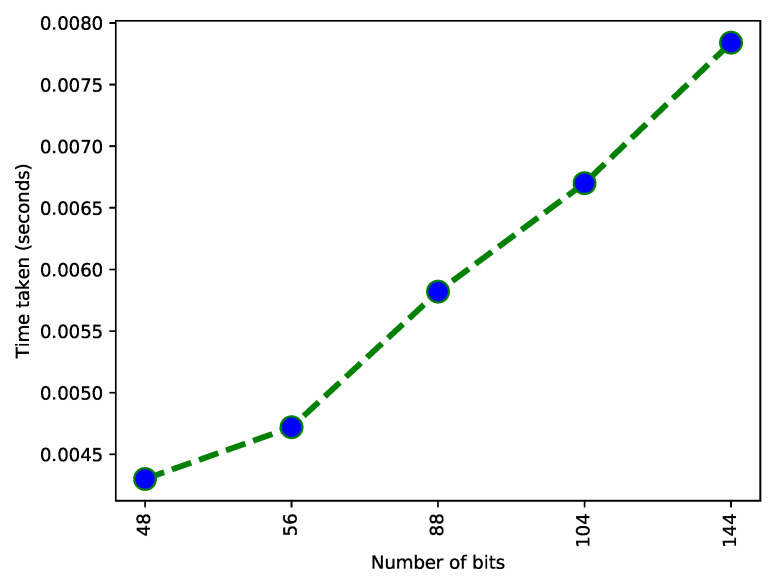
Time taken for the conversion of bits.

Figure 7 shows a relation between time and data size. This data are based on character strings of different sizes. These strings are the number of bits; each bit takes some time to convert in the hash. This graph shows an exponential trend of increasing time because when the number of bits increases, the time for hashing increases. The number of bits and time are proportional. One character uses 8 bits for storage. This hashing technique is used to avoid data redundancy. This figure is the validation of L5 as shown in Table 2.

**Figure 8:**
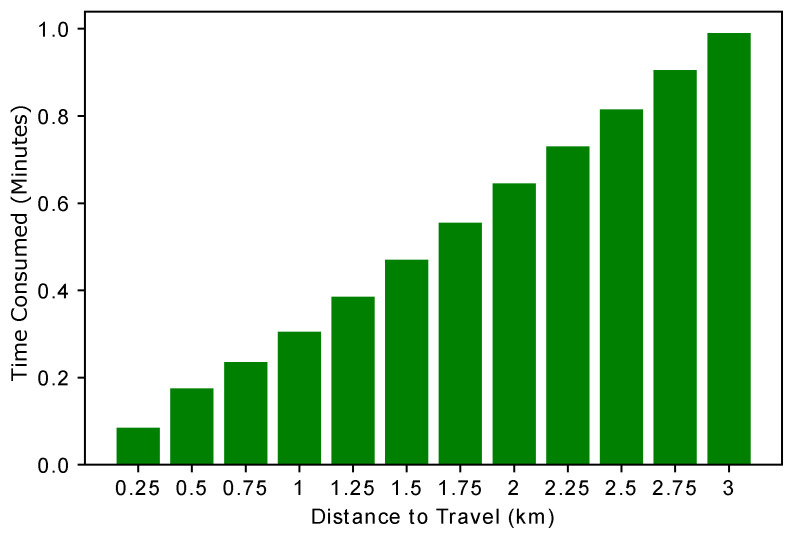
Time taken to travel a certain distance.

Figure 8 shows the relationship between the time and distance required to reach the destination. Whenever an EV wants to buy energy from a charging station, it must cover some distance to reach the charging station. While covering this distance, the EV consumes some time. The time calculation helps the EV set its charging schedule according to the time required to reach the charging station. However, we have considered only the charge consumption cost and time, while the other factors are neglected in our case. A linear trend is observed in Figure 8, which means that as the distance between the EV and the charging entity increases, more monetary cost is required to reach the charging station. This figure is the validation of L1 as shown in Table 2.

**Figure 9:**
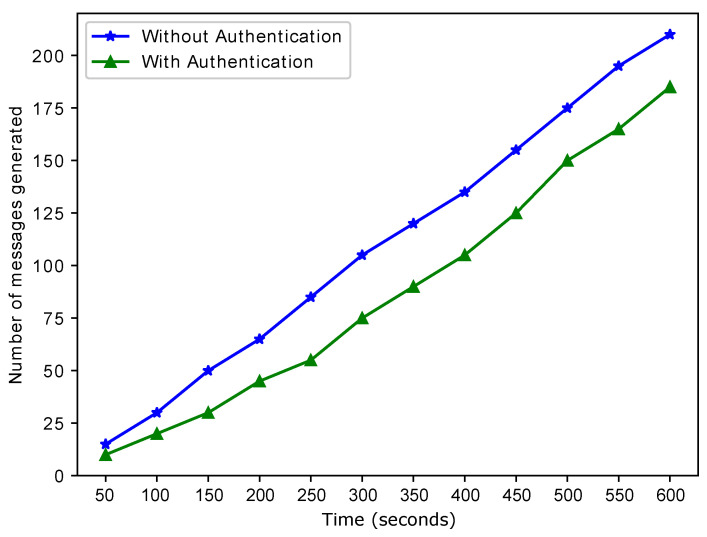
Time versus the number of generated messages.

The selection of the charging entity depends on the time taken to reach that entity. If the time taken to reach the charging entity is much greater than a specific value, then that entity is discarded, and a closer entity is selected. The time taken to reach the charging entity can be affected by external factors such as road congestion, obstruction on the road, etc. However, these external factors are not considered. Figure 9 shows the difference between the number of messages generated by the authenticated EV and the messages generated by both authenticated and unauthenticated EVs. The elimination of unauthenticated messages leads to a reduction in the storage required to store the messages. The presence of unauthentic EVs in the VN leads to the generation of fake messages. When the number of malicious EVs increases, the number of fake messages also increases. Generating many fake messages leads to data redundancy, and hence, data storage issues. Removing unauthenticated vehicles from the network is required to tackle the message storage issue and increase network security. This figure is the validation of L4 as shown in Table 2.

**Figure 10:**
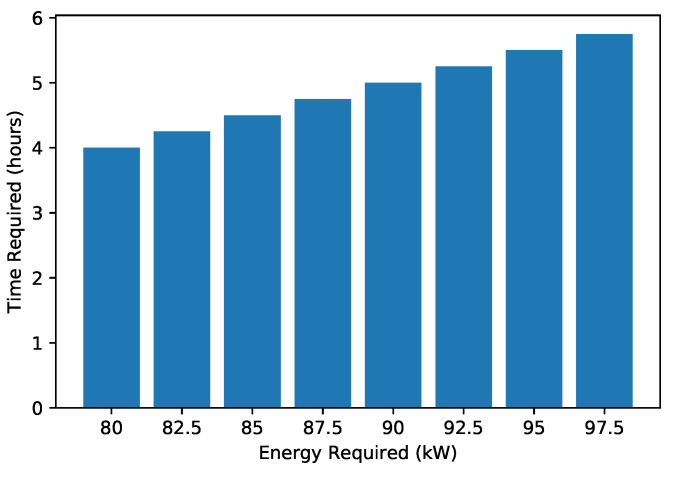
Time versus energy required.

**Figure 11:**
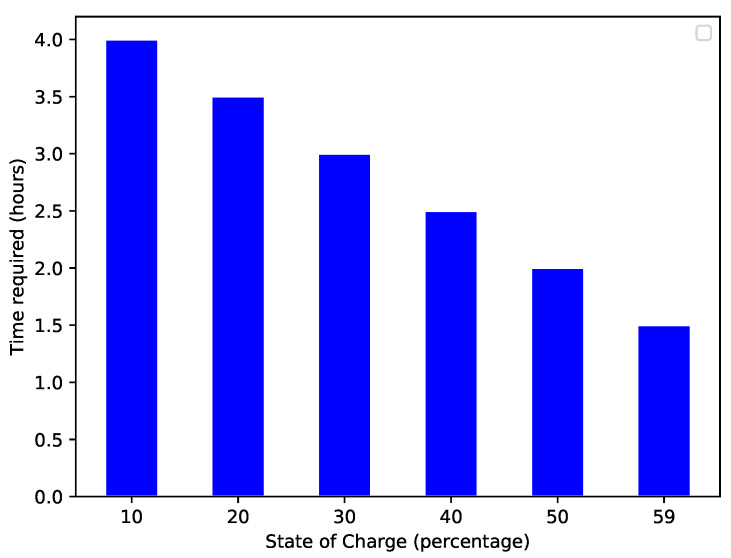
Present SoC of the EV and time required for charging.

**Figure 12:**
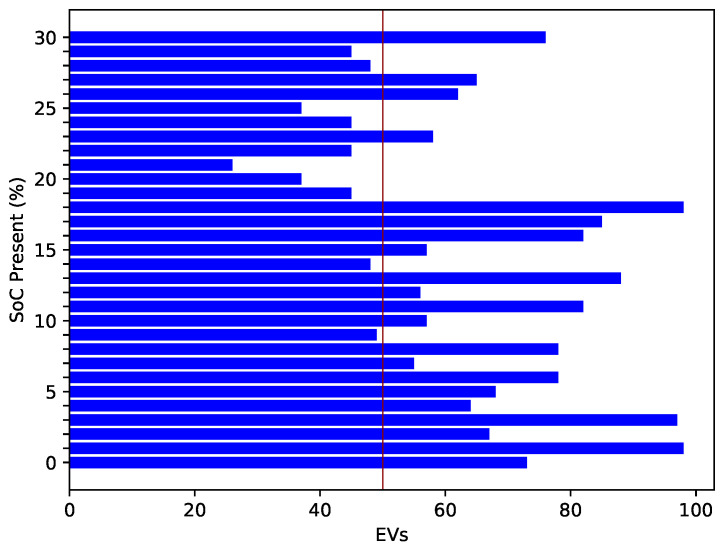
Present SoC of EV.

Figure 10 shows the required energy to reach the charging station. A high amount of energy is commonly required to cover a long distance. There is a direct relationship between the amount of energy and distance. Figure 11 shows the EV’s present SoC and the time (in hours) that an EV requires to become fully charged. When an EV has a high SoC, it requires less time to charge and vice versa. The present SoC and time taken to charge are inversely proportional; therefore, the graph shows a decreasing trend. The total charging capacity of an EV is 100%; required time for charging is calculated by subtracting the present SoC percentage from 100. Figure 12 shows the trend of the present charging state and the number of vehicles present in the network. Figure 12 depicts an inverse relationship between the time the EV takes for charging and the present SoC.

**Figure 13:**
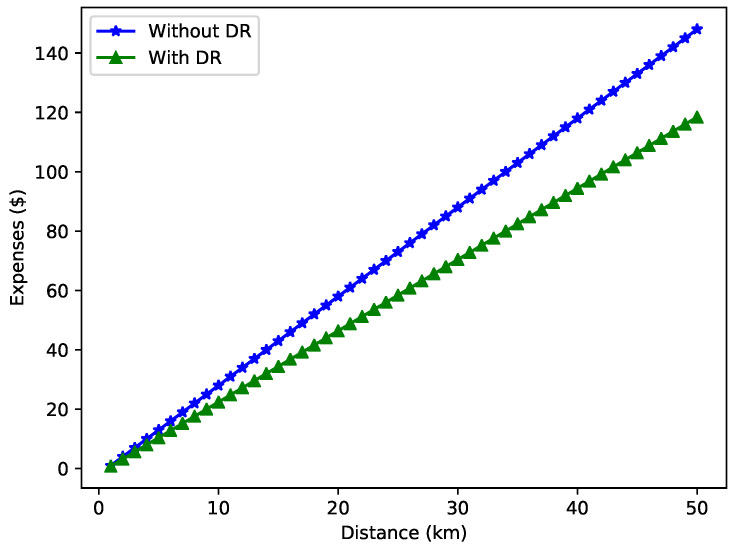
Expenses incurred with DR.

Figure 13 compares the expense incurred with and without DR while traveling to the selected charging entity. It is visualized that DR helps minimize expenses to a great extent. Initially, for small distances, the difference is not much. However, as the distance increases, the difference becomes large because of the high charging energy consumption. Therefore, EVs tend to apply the DR mechanism to adjust the load curves.

## 5. Security Analysis of the Proposed Smart Contract

**Figure 14:**
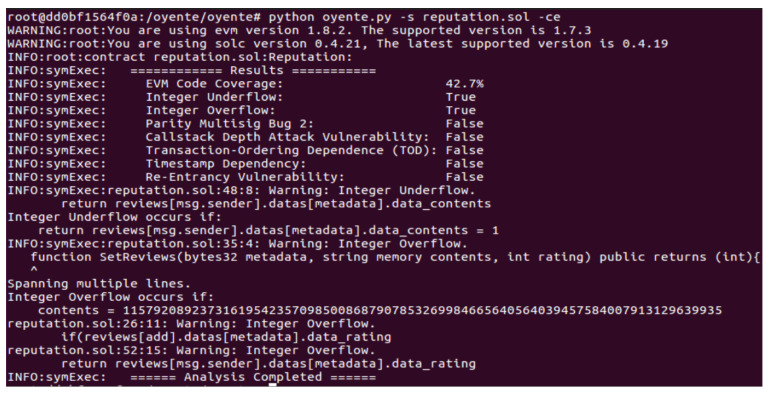
Security analysis of the proposed smart contracts.

This section consists of the analysis of the proposed smart contract and the finding of related vulnerabilities. Figure 14 shows the security analysis of the proposed smart contract through Oyente. Oyente is used for the analysis of smart contracts. It is an open-source tool developed by [52]. It analyzes smart contracts using symbolic execution techniques based upon the execution of step-wise functions [53]. Oyente software provides a flexible environment that works directly with an Ethereum Virtual Machine (EVM) and does not require access to high-level representations such as Solidity, Serpent, etc. [54]. Moreover, it is also used to analyze smart contracts against the following major vulnerabilities and attacks. However, we have not tackled these attacks in our proposed system.

- Re-entrancy vulnerability
- Timestamp dependency
- Callstack depth vulnerability
- Transaction ordering dependency
- Parity multisig bug
- Integer overflow
- Integer underflow

Figure 14 shows the security analysis of the smart contract involved in the proposed model. The figure shows that the outputs of almost all results in the analysis report are “False”, which indicates that the proposed smart contracts are robust against many well-known vulnerabilities. Many false results mean the proposed model is secure and robust against these attacks. However, the smart contract faces two types of vulnerabilities: integer overflow and integer underflow. Integer overflow occurs when the quantity of integers used in a specific function exceeds the defined limit, whereas integer underflow occurs when the quantity of integers is less than a threshold value required for the function’s execution.

### 5.1. Security Features

In this section, we discussed the solutions of our security model and how it deals with security threats and ensures system security. The proposed solution consists of blockchain features. These features are decentralization, integrity, non-repudiation, trust and availability. This system is protected against man-in-the-middle (MITM) and replay attacks.

#### 5.1.1. Integrity

Integrity is an important feature that is used to ensure that there have been no data modifications. The immutability of blockchain ensures data integrity and exchanges messages between all participants and generates logs and events.

#### 5.1.2. Availability

Availability ensures the deployed smart contract in the blockchain is always available for all participants. This ensures that all present network services are always available for the users. It protects the system from denial-of-service (DoS) attacks. All transactions are stored in the distributed Ethereum ledger; therefore, there is no fear of hacking, failure or compromise. The Ethereum ledger is highly robust against DoS attacks because thousands of mining nodes protect it.

#### 5.1.3. Confidentiality

The confidentiality requirement is achieved using a private/permissioned blockchain such as private Ethereum and Hyperledger. The proposed system consists of a consortium blockchain.

## 6. Blockchain-Based Attacker Model

Blockchain networks are generally considered secure, immutable and scalable networks. However, some attacks can harm the network because of its security level. The security level of a blockchain network is directly proportional to the number of miners. The security level increases with an increased number of miners.

A blockchain network can be attacked by several attacks, such as Sybil, routing, DDOS and double-spending attacks.

### 6.1. Double-Spending Attack

In this attack, digital currency can be spent twice. Unlike physical currency, a digital token can be easily modified through a potential flaw. Therefore, it can easily be falsified and duplicated. This attack occurrs when a digital currency is stolen in a disrupted network. In energy trading systems, attacks are not only security threats; they also result in financial loss. A double-spending attack can occur during EV and charging station transactions in the proposed system. The attack model introduced by Satoshi Nakamoto is similar to Rosenfield’s attack model [55]. The parameters used in both models have the same definitions and use similar notions. The parameters used in the proposed model are given below.

- $C_{N}$: a catch-up function that shows the probability of the fake longer chain published by the attacker.
- *T*: a random variable that shows the time needed for mining.
- $P_{N}$: a potential progress function. It shows the probability of mining by an attacker.
- *m*: in the double-spending attack, attackers mine the *n*th block and the honest nodes mine the *m*th block.
- *z*: *z* is the initial disadvantage of the attacker.
- *x*: the computation power available in the network.
- *q*: the probability that the attacker will mine the block before the honest miner when both miners start mining simultaneously. In other words, it can be said that *q* is the proportion of the attacker’s computation power. The value of *q* belongs to [0, 1], and q=p−1.
- *n*: the number of mined blocks.
- *t*: the time advantage of the attacker.
- *K*: the number of confirmations needed to declare a block and the transaction as valid. This parameter depends upon the seller and not the network. The value of *K* belongs to the set of natural numbers N.
- τ: the average time required by the honest and attacker nodes for block mining. The value of τ belongs to the set of real numbers $R_{>0}$.

### 6.2. Mathematical Formulation

The mathematical formulation of double spending is described in this section. These equations are based on [55]. The probability of a double-spending attack is related to the mining time of a block. The attacker mines block 1 to block *n* and ends up with a difference of K−n blocks. This is given in Equation (3).

(3) $$\begin{array}{cc} DS_{N}\left(q,K\right) & =\sum_{n=0}^{+\infty }P_{N}\left(q,K,n\right)C_{N}\left(q,K-n-1\right)\\ \; & =1-\sum_{n=0}^{K}P_{N}\left(q,K,n\right)\left(1-C_{N}\left(q,K-n-1\right)\right) \end{array}$$

where C(q,z) is given as

$$C\left(q,z\right)\left\{\begin{array}{c} {\left(\frac{q}{p}\right)}^{z+1}\;,\;if\;q<0.5\wedge z>0\\ 1\;\;\;\;,\;otherwise \end{array}\right.$$

In the above equation, *q* identifies the attacker’s computational power, and *p* shows the probability of less computational power of an attacker, where p=1−q calculates the computational power of an attacker in the network. The probability that the attacker is successful in mining the block before the honest block is given using Equation (4).

$$P\left(T_{q}<T_{p}\right)=\int_{0}^{\infty }P\left(T_{q}=x\right)P\left(T_{p}>x\right)dx$$

$$\;\;\;\;=\int_{0}^{\infty }\frac{q}{\tau }e(\frac{-q}{\tau }x)e(\frac{-p}{\tau }x)dx$$

$$=q\int_{0}^{\infty }\frac{1}{\tau }e(\frac{-1}{\tau }x)dx$$

(4) =q

The attacker’s potential progress function is defined using Equation (5).

(5) $$P\left(q,m,n,t\right)=\sum_{z=0}^{n}a\left(q,t,z\right)P_{N}\left(q,m,n-z\right)$$

where

$$a\left(q,t,n\right)=\left\{\begin{array}{c} 1\;\;\;\;\;\;,\;if\;t=n=0\\ 0\;\;\;\;\;\;,\;if\;t<=0\\ \frac{{\left(qt\right)}^{n}}{n!}e^{-qt},\;,\;otherwise \end{array}\right.$$

**Figure 15:**
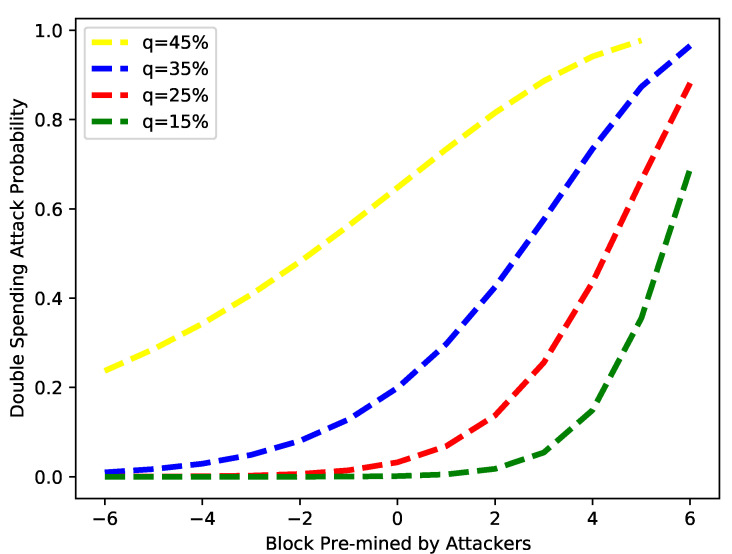
Probability of double-spending attack vs. block advantage.

The impact of a double-spending attack in the proposed work is evaluated using the time advantage, computing power, and the number of pre-mined blocks. These pre-minded blocks are mined by the attacker and are known as negative blocks. If the number of negative blocks is increased in the network, then the probability of a double-spending attack is increased. In Figure 15, the number of pre-mined blocks is used as an input. The results are obtained for different values of *q*: 15%, 25%, 35% and 45%. It is observed from the figure that for *q* less than 30%, the probability of a successful double-spending attack begins after the creation of some blocks, at which point this probability increases to 35%. For values of *q* greater than 40%, the double-spending attack can occur after creating just a few blocks. This means if the value of *q* increases, the probability also increases, and after attackers control the network, the chances of double-spending attacks are increased greatly. Probability values close to zero show that a double-spending attack will be unsuccessful, while values closer to one show a high success rate.

**Figure 16:**
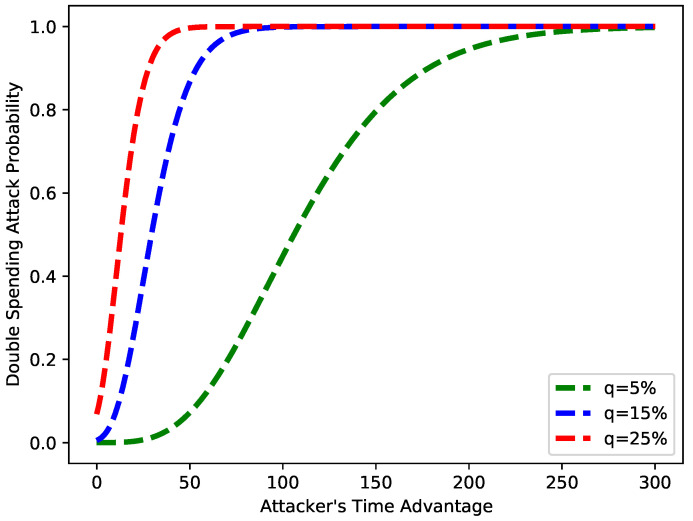
Probability of double-spending attack vs. time advantage.

Figure 16 depicts the probability of a double-spending attack versus the time taken to launch the attack. From the figure, it is obvious that as the value of *q* increases, the time required for a double-spending attack is lessened. The figure shows that when *q* is 5%, the attack starts happening after 50 s, and when *q* is 25%, the attack happens in milliseconds. This means when the number of fake blocks increases, the probability of an attack also increases.

**Figure 17:**
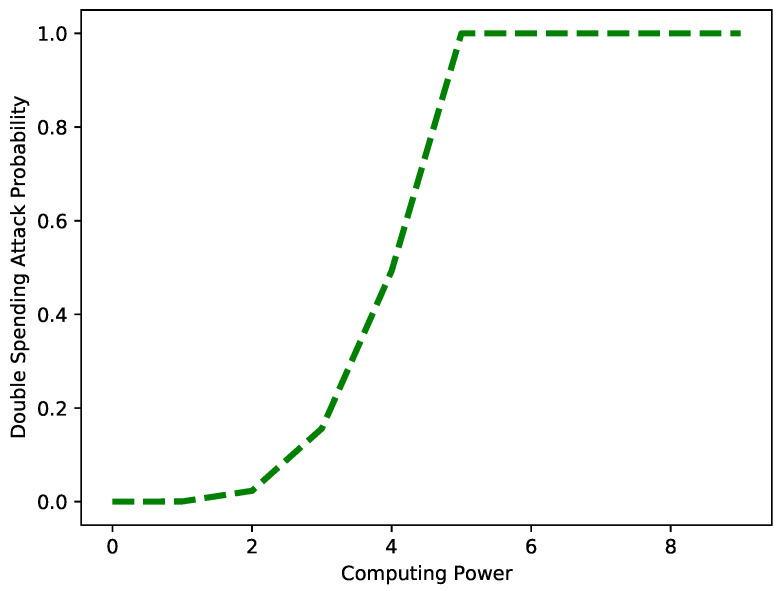
Probability of double-spending attack vs. computing power when *q* = 60%.

Figure 17 depicts the probability of a double-spending attack against the computing power of an attacker. The figure depicts that as the computing power of the attacker increases, the probability of the attack also increases. The increased computing power means the attacker has sufficient time to mine a new block. The results are obtained for *q* = 60%.

### 6.3. Replay Attack

In a replay attack, the attacker saves sensitive information from the network and uses it after some time to gain incentives. It is also called a playback attack, in which a malicious entity repeats a valid transaction to gain financial incentive. This attack can also be used to gain access to valid credentials of the network. A replay attack can occur between EVs and charging stations in the proposed system. When a transaction occurs between an EV and a charging station, the attacker entity saves the transaction’s data and uses them on other charging stations. In the proposed system, we set a specific time period to update the reputation of EVs. If the reputation value of the EV is older than a specific threshold, the transaction is considered malicious.

**Figure 18:**
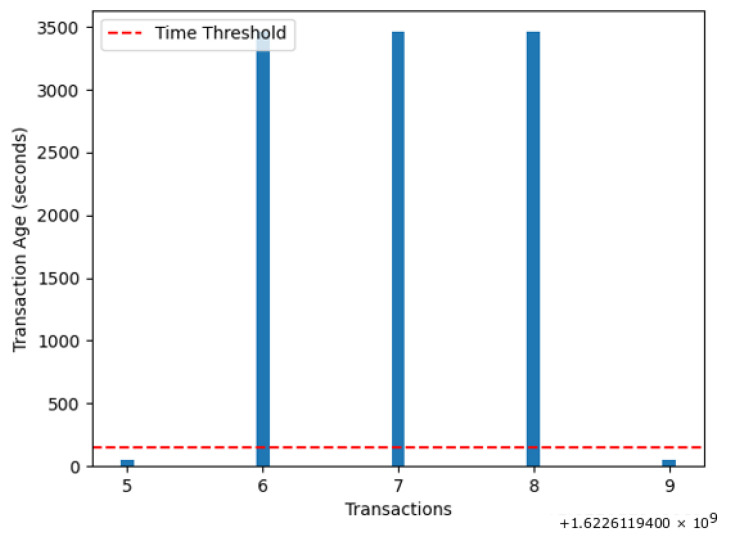
Number of transactions versus transaction age.

Figure 18 shows the transaction age of both honest and fake ids. The attackers created fake IDs, which are shown on the upper side of the red line. The bars shown in blue color, which are lower than the red line, are the transactions performed by the honest nodes. The red dotted line shows the threshold limit of transaction age, which is set as 150 in our case. It can be observed from the figure that the fake transactions cross the threshold limit.

On the other hand, the transaction age of honest transactions is almost half that of the malicious transactions. This 1:2 proportion shows the occurrence of a replay attack.

## 7. Conclusions

In VEN, a novel charging algorithm is proposed with moderate cost. The proposed model comprises machine learning, blockchain and DR. Moreover, the proposed system consists of consortium blockchain, energy trading, EVs, charging stations, DR and a branching mechanism. The incorporation of blockchain technology promotes security and ensures secure data storage, immutability and transparency in the proposed work. In the underlying work, the coordination between different EVs, charging stations and aggregators is also done securely and efficiently. The proposed work helps solve the communication issues in VEN.

Furthermore, the most commonly used machine learning algorithm, KNN, is used in the proposed work to find the nearest charging station, which reduces resource consumption and computation power. EVs communicate with charging stations through aggregators to fulfil their energy requirements and pay in the form of cryptocurrency. The time required to charge the vehicles depends upon the charging station’s distance, and the SoC value is also calculated in this work. The proposed model is more efficient than present work, as a branching concept for data is used, which reduces computational delay and solves storage issues. The branching mechanism is also involved in the proposed system to deal with the complexity of intensive data. While performing transactions, data redundancy is also resolved in the proposed work via SHA-256 hashing. We used Oyente to analyze the bugs and vulnerabilities in the proposed smart contracts, and we also checked the robustness of the network against double-spending and replay attacks. The analysis of smart contracts also shows that our system improves the security and privacy of transactions.

## 8. Future Work

In this paper, we worked on optimal energy usage in EVS. A novel algorithm is proposed for EVs to find the nearest optimal charging station. The results of our proposed scheme outperform and show that the EVs use less computational power. Previously in [48], the authors worked on energy trading between EVs and charging stations; however, they included a third party, which may cause security issues and used a cloud for storage, which creates a single point of failure. Therefore, we did not include any third party in our proposed system and used IPFS for data storage. However, in the future, we will compare our proposed model with other charging schemes with the same parameters and implement our proposed model in a real-time scenario. Moreover, a novel variable pricing scheme will be used in vehicular systems, which will allow users to charge their vehicles at affordable prices.

## Figures and Tables

**Table 1:** Related work.

Reference	Year of Publication	Addressed Limitations	Proposed Solutions	Limitations
[12]	2019	Power supply	A dynamic complex energy network	Did not consider centralized energy storage points
[13]	2018	Inefficient energy management	A blockchain-based scheme for management of charging piles	Maintenance of the system is expensive
[14]	2017	Inefficient charging strategies and trust issues	A consortium blockchain system	Requires high mining cost
[15]	2020	Discussed different charging infrastructures and strategies in smart cities	Analysis of different charging strategies	None
[16]	2019	Insecure energy trading	Used a dynamic pricing strategy and a reverse-auction mechanism	Centralized grids
[17]	2019	Trust issues among EVs	A decentralized trust management system based on blockchain	Lacked both trust management and privacy preservation
[18]	2019	Security issues in energy trading	An incentive scheme based on blockchain	Malicious entities are not considered
[19]	2019	Secure and efficient data trading using consortium blockchain	A consensus mechanism based on pre-selected nodes	Increased energy consumption because a large number of iterations is involved during the process
[20]	2017	Inefficient charging of PHEVs and communication issue	Energy trading mechanism for (PHEVs)	Balancing of energy is not considered
[21]	2018	Introduced a new concept related to EVs in energy markets: G2V and V2G	Proves that an energy grid is an advantageous entity	Leads to environmental pollution.
[22,23]	2018, 2020	Security analysis of the Brooklyn microgrid network	An encryption scheme is used for the security of transactions	Malicious operators and selfish mining are not considered
[24]	2018	VN insecure energy management	A decentralized security model	Privacy of EVs is not considered
[25]	2020	Energy management problems	Used a deep CNN model with blockchain for energy management	Complexity is an issue
[26]	2019	High delay in service response and lack of trust	A blockchain-based intelligent, secure autonomous transportation system	Did not consider storage issues
[27]	2018	Security issues in SDN	A novel hybrid architecture network	Did not consider the efficient deployment of edge nodes
[28]	2022	Addressed the controller selection problem	Analytical Network Decision-making Process (ANDP)	Did not consider scalability issues
[29]	2018	Security threats and trust issues	An intelligent vehicular network based on blockchain	The comfort of vehicle operators in a hassle-free network is not considered
[30]	2019	Storage and security issues	A blockchain-based decentralized, distributed and secure storage management scheme	Channels are unreliable during vehicle communication
[31]	2019	Trust issues	A decentralized trust-management system based on blockchain	Message validation delay is increased
[32]	2018	Uncertainty and randomness of the charging and discharging of EVs	A decentralized power-trading model	High implementation cost
[22]	2018	Integrated blockchain with EVs for security purposes	Designed a multi-blockchain architecture	Multi-blockchains become expensive
[33]	2017	Security and privacy problems of energy trading networks	A consortium blockchain-based secure energy trading system	Requires high cost to maintain an energy blockchain with IIoT nodes
[34]	2019	Blockchain technology is integrated with edge computing in a VN	A contract theory-based incentive mechanism	The given approach requires further discussion
[35]	2019	Insecure energy trading and malicious activities	Smart-contract-based secure energy blockchain system	Privacy issue is not resolved
[36]	2019	Deficiencies in dealing with the profits made by charging stations	Proposed an optimal pricing scheme for charging EVs	Coordination issues

**Table 2:** Mapping of problems with proposed solutions and validation results.

Addressed Limitations	Proposed Solutions	Results and Validations
**L1**: Vehicles use high computational power and resources to find an optimal charging station.	**S1**: Finds the shortest distance by using a machine learning algorithm	**V1**: Figure 10 depicts the expenses used by an EV according to the travelling distance.
**L2**: The energy sector faces new challenges such as imbalanced load supply, fluctuations in voltage level and load shedding.	**S2**: The integration of DR in VNs becomes necessary as it helps to manage the load supply and efficiently reduce the peak load.	**V2**: Figure 13 depicts the load consumption with and without using DR.
**L3**: Multiple vehicles send requests to the aggregator simultaneously. Therefore, selecting the desired vehicle becomes difficult in the network/system.	**S3**: A reputation mechanism is proposed for the preferred selection of EVs.	**V3**: The validation of this reputation mechanism is shown in Figure 4 as the deployment of a smart contract that assigns reputations to EVs.
**L4**: Malicious operators in energy markets are threats to network privacy and security through exploitation, e.g., privacy leakage and node impersonation.	**S4**: To resolve this problem, we use authentication.	**V4**: Figure 9 depicts the number of authentic and unauthentic messages generated by EVs.
**L5**: Data redundancy issues exist.	**S5**: A SHA-256 hashing algorithm is used to remove/detect data redundancy. Hash values of newly uploaded data are compared with the hash values of existing data to find duplication.	**V5**: Figure 8 shows the encryption of character strings into bits.

## Data Availability

The data used in this research can be obtained from the corresponding authors upon request.

## Abbreviations

The list of acronyms is given in the following:

**Acronym**	**Description**
$B_{i}^{cap}$	Battery capacity
$E_{dem}$	Energy demand
$E_{i}^{av}$	Available energy at charging station
$E_{i}^{th}$	Threshold for charging
$E_{i}^{pr}$	Present energy in EV
$Loc^{j}$	Location of charging station
$P_{j}^{s}$	Energy price
$SoC{pr}_{j}$	State-of-charge
$T_{i}^{req}$	Time required for charging
$C_{N}$	Catch-up function
*K*	Confirmation number to declare a block
*m*	Block mined by the honest nodes
*n*	Block mined by the attacker
$P_{N}$	Potential progress function
*q*	Attack probability
*T*	Time required for mining
*t*	Time advantage for the attackers
τ	Average time to mine a block
*x*	Computational power available in the network
*z*	Attacker’s initial disadvantage
